# Prenatal assessment and pregnancy outcomes of foetal low-lying conus medullaris using 3D ultrasound

**DOI:** 10.1186/s12884-022-05244-3

**Published:** 2022-12-23

**Authors:** Baihua Jing, Huijing Zhang, Yu Sun

**Affiliations:** The Department of Obstetrics and Gynaecology, Peking University First Hospital, Beijing, China

**Keywords:** Prenatal, Low-lying conus medullaris, 3D ultrasonography, Pregnancy outcome

## Abstract

**Objectives:**

This study aims to follow up on low-lying conus medullaris (CM) cases and explore the correlation between the CM location and the final prognosis.

**Methods:**

We retrospectively collected 37 cases diagnosed with low-lying CM during pregnancy in the Peking University First Hospital from January 2019 to December 2020. The location of CM was confirmed by 3D ultrasonography, and clinical data, including postnatal outcomes, were recorded. When the conus medullaris was below L3 (excluding L3), it was diagnosed as low-lying conus medullaris, regardless of gestational age. The short-term postnatal outcome included assessment of symptoms and signs of motor and sensory neuron dysfunction.

**Results:**

The average gestational weeks of low-lying diagnosis was between 23 and 24 weeks. Among 37 cases, nine (24.3%) were complicated with spine dysraphism (3 cases of open spina bifida, 6 cases of tethered cord syndrome). Apart from 7 cases of pregnancy termination, the remaining 30 live births had a good prognosis in the short term, though 5 out of 6 cases of tethered cord syndrome underwent surgical release. The mean location of cases of open spinal dysraphism (*n* = 3) and those of closed dysraphism/tethered cord syndrome (*n* = 6) was at Lumber vertebra 5 (L5) and between L5 and Sacral vertebra 1 (S1), respectively, which showed statistical significance compared with the postnatally normal group. When we set Lumber 4.25 as the cut-off value to predict the diagnosis of spine dysraphism (mainly involving open spinal dysraphism and closed spinal dysraphism/tethered cord syndrome), the sensitivity was 66.7. At the same time, the specificity was 96%, along with the area under the curve (AUC) at 0.877.

**Conclusion:**

The second trimester finding of low CM is associated with spinal defects, mainly open spinal dysraphism and closed spinal dysraphism/tethered cord syndrome. Careful assessment of the fetal spine should be considered especially when the location of CM is lower than L4.

## Introduction

With advances in ultrasound technology and the growing understanding of foetal disorders, the implementation of prenatal ultrasound for foetal central nervous system (CNS) examination has become increasingly important. The International Society of Ultrasound in Obstetrics and Gynaecology (ISUOG) has recently updated its guidelines on foetal central nervous system ultrasound, suggesting that the conus medullaris helps determine the normality of the lumbosacral spine [1] and can help to detect neural tube defects. The most common neural tube defects are spina bifida and meningocele [2, 3] and closed spinal dysraphism. These are associated with lipoma, diastematomyelia, epidermoid cysts, and dermoid cysts [4].

A low-lying conus medullaris (CM) refers to the condition with conus medullaris below L3 (excluding L3), regardless of gestational age [1]. It could lead to some clinical symptoms, such as motor and sensory dysfunction of lower extremities, urination and defecation function disturbance. As a result, diagnosing "low-lying CM by prenatal ultrasound has become increasingly necessary in recent years. There are few studies on the correlation between ultrasonic manifestations during pregnancy and postnatal pregnancy outcomes. This study aimed to provide our experience and clinical outcomes of foetuses prenatally diagnosed with low-lying conus medullaris.

## Data and methods

### Study subjects

As a retrospective observational study, 37 patients diagnosed with low-lying CM on ultrasound were collected from January 2019 to December 2020 at the Department of Obstetrics and Gynaecology, Peking University First Hospital, Beijing, China. All these patients received routine prenatal care and gave birth in this institution.

### Instruments and study methods

**Fig. 1:**
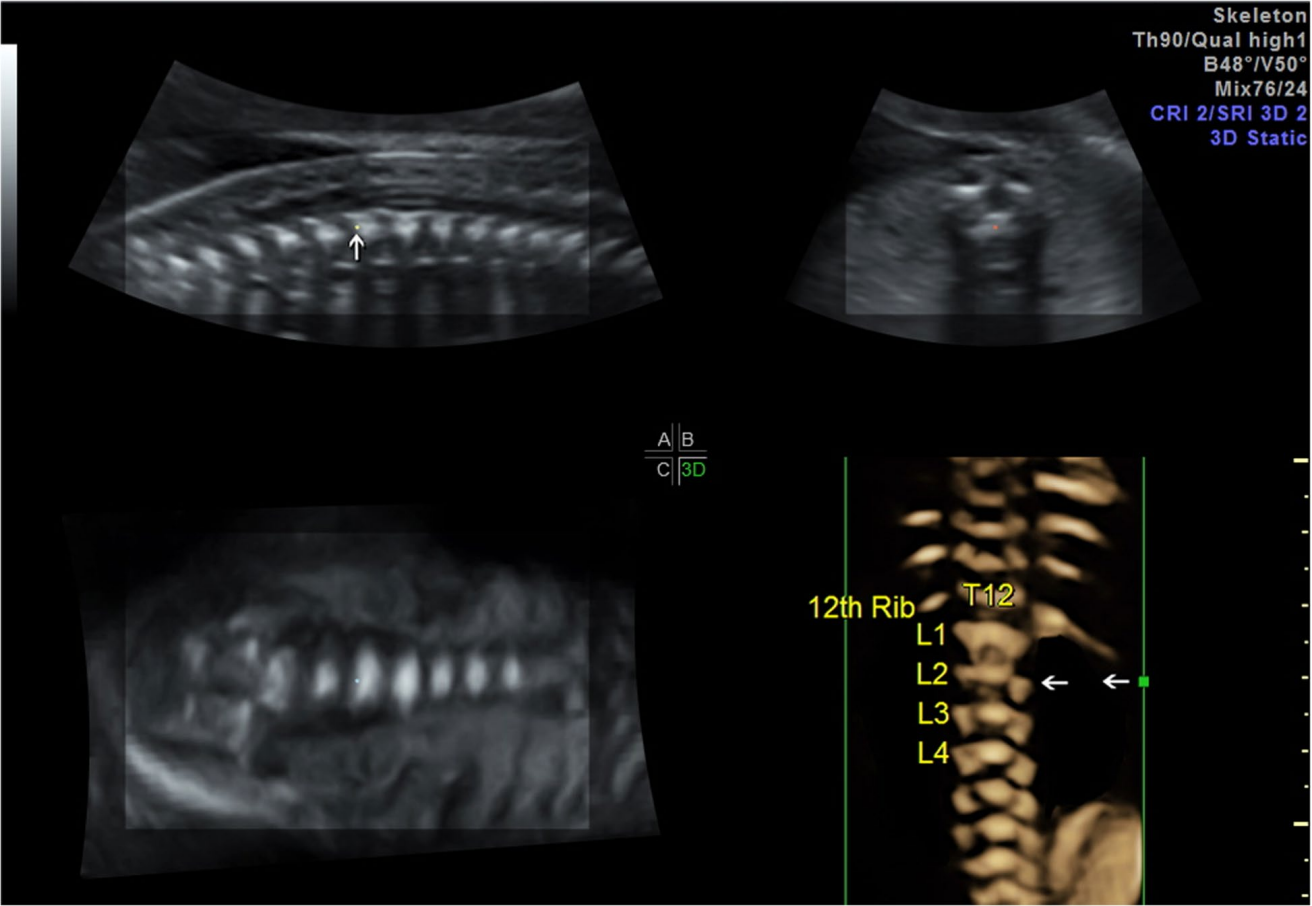
The (**A**) plane was obtained at the sagittal plane of the foetal spine to clearly show the end of the conus medullaris, and the reference point was placed here (Fig. 1, white dot). The (**B**) plane was the view of the transverse spine, while the (**C**) plane was the coronal spine view. The (**D**) plane was the 3D reconstruction of the spine. The green dot in the D plane corresponded to the white dot in the A plane

- The spine was assessed at the transverse, sagittal, and coronal planes per the guidelines on foetal central nervous system ultrasound published by ISUOG [1]. Since a three-dimensional (3D) scan was not routine while examining the foetal spine, the radiologists only implemented the 3D scan if there was a susception of low-lying conus medullaris during a two-dimensional (2D) scan. To obtain a high-quality 3D volume of the foetal spine, we used intermediate-frequency transducers (4–8 MHz) to identify the position of CM. The acquisition angle ranged between 45° and 60° under skeleton mode [5]. The study was conducted according to the guidelines of the Declaration of Helsinki and approved by the Institutional Review Board of Peking University First Hospital (protocol code 2013[572]).
- Definition of Low-lying CM: When the CM was below L3 (excluding L3), it was diagnosed as low-lying CM, regardless of gestational age [1].
- Foetal CM Position Examination: During the 3D examination, we first displayed the median sagittal section of the spine to obtain a clear view of CM (Fig. 1, white arrow). Then we instructed the pregnant woman to hold her breath and initiated a 3D volumetric scan (Fig. 1). We adjusted the parameters so that the D plane clearly showed the spine and ribs. We determined the position of the 12th thoracic vertebra based on the location of the 12th rib. Then we counted the lumbar vertebras (L) 1, 2, and 3 downwards until they were at the level of the reference point (Fig. 1, green dot). If the CM was between the 3rd and 4th lumbar vertebrae (L3-L4), this was recorded as 3.5.
- Clinical Data Collection: The clinical baseline data of all pregnancies were collected: age, gestation week when low-lying conus medullaris was firstly detected, changes of CM position during pregnancy, presence of other non-spinal structural abnormalities, pregnancy outcome, gestational age at birth, and birth weight. The short-term postnatal follow-up by telephone ran until August 1, 2021, which included an assessment of symptoms and signs of motor and sensory neuron dysfunction [6].
- The primary outcome of this study included: (1) clinical characteristics of low-lying CM cases; (2) the correlation between the CM location and spinal defect; (3) the final prognosis of low-lying CM.
- Statistical Methods: The clinical data of patients with foetal conus medullaris found during pregnancy were analysed descriptively. The mean (± standard deviation) was calculated for measurement data, and the number of cases (percentage) was reported for count data. The conus medullaris positions of the spinal defects and normal spine groups were compared by t-test, and the differences were considered statistically significant with P < 0.05. Spine dysraphism was diagnosed according to clinical criteria. We used receiver operating characteristic (ROC) curve analysis to explore the diagnostic value of the conus medullaris position using 3D. The cut-off values were selected according to sensitivity and specificity. Statistical analysis was performed with IBM SPSS 25.0 software.

## Results

### General information

As shown in Table 1, the average age of 37 pregnant women was 31.9 years, and the gestational age when low-lying conus medullaris was first detected was 23–24 weeks. Nine cases of spinal anomalies were confirmed postnatally, with 3 cases of open spinal dysraphism and 6 cases of closed spinal dysraphism or tethered spinal cord. Seven low-lying CM cases (18.9%) were complicated with non-spinal structural anomalies. Two (2/7, 28.6%) were finally confirmed with spinal dysraphism. Thus, the rate of non-spinal structural defects in the group of spinal defects (2/9, 22.2%) was similar to that of the normal spine group (5/28, 17.9%).

**Table 1:** Demographic Data and Pregnancy Characteristics of Study Population

Clinical Data	Number
Age	31.89 (± 4.46)
Singleton	33 (89.2%)
Twin	4 (10.8%)
Gestation week at which “low-lying conus medullaris” was first detected	23.81 (± 3.84)
Spinal anomalies	
Open spinal dysraphism	3 (8.1%)
Closed spinal dysraphism/Tethered spinal cord	6 (16.3%)
Dermatomal sinus	1 (2.7%)
Lipoma	1 (2.7%)
With other foetal structural abnormalities	7 (18.9%)
Complicated with spinal dysraphisms	2
Bilateral Ventriculomegaly and bilateral hydronephrosis	1
Cleft lip and palate	1
Not complicated with spinal dysraphisms	5
Absent nasal bone and hyperechogenic bowel^a^	1
Left renal agenesis and ventricular septal defect^a^	1
Anal atresia	1
Non-visulisation of gallbladder	1
Short long bones & Foetal growth restriction^a^	1
Pregnancy Outcome	
Live birth	30 (81.1%)
Termination of Pregnancy (TOP)	7 (18.9%)
Non-spinal abnormality	3
Absent nasal bone and hyperechogenic bowel^a^	1
Left renal agenesis and ventricular septal defect^a^	1
Short long bones & Foetal Growth restriction^a^	1
Open spina bifida	3
Genetic abnormality arr[GRCh37]16q24.1(84850607_85984462) × 3	1

^a^Cases who had an amniocentesis

As for the indications for 7 cases that underwent amniocentesis, three were due to multiple anomalies and these three pregnancies ultimately terminated despite normal genetic results (Table 1). Another three indications were advanced age (one chose to terminate the pregnancy due to abnormal genetic results, while the other two had normal genetic results and live births). The last one's indication was low-lying conus medullaris and maternal request with a final normal genetic result and live birth.

Regarding the reason for TOP, three were due to multiple anomalies, three were due to open spina bifida, and one was due to genetic abnormality. 

### Spinal anomalies with the conus medullaris at a different location

Table 2 demonstrated spinal abnormalities at different conus medullaris locations. For the foetuses confirmed with spinal dysraphism, the position of conus medullaris was below L4.

**Table 2:** Position of conus modularis and final postnatal diagnosis

	Number of cases (%)	Open spinal dysraphism	Closed spinal dysraphism/Tethered spinal cord
L3-L4	10 (27.0%)	0	0
L4	20 (54.1%)	1	2
L4-L5	2 (5.4%)	1	1
L5	2 (5.4%)	0	1
S1-S2	1 (2.7%)	1	0
S2	1 (2.7%)	0	1
S3	1 (2.7%)	0	1

### Comparison of CM location between foetuses with spinal defects and those with a normal spine

In this part, we marked the lumbar vertebra as one at position L1, two at L2, and so forth. The vertebral positions of the conus medullaris in the spinal defects group were compared with those in the normal spine group, respectively (Table 3). The average maternal age of the open spinal bifida group and closed spinal dysraphism was 36.33 and 31.00, compared to that of the normal spine group (31.61) with no statistical difference. The average CM position was at the L5 level in the open spinal bifida group, between L5 and S1 in the closed spinal dysraphism and tethered cord syndrome group, and above L4 in the normal spine group. The differences between groups were statistically significant.

**Table 3:** Comparison of CM Position between the spinal defects group and normal spine group

	Conus medullaris position, mean (standard deviation)	*P*
Open spinal dysraphism (*N* = 3)	5 (1.32)	< 0.001
Closed dysraphisms/Tethered cord syndrome (*N* = 6)	5.42 (1.69)	< 0.001
Normal spine (*N* = 28)	3.8 (0.33)	

### ROC curve analysis

**Fig. 2:**
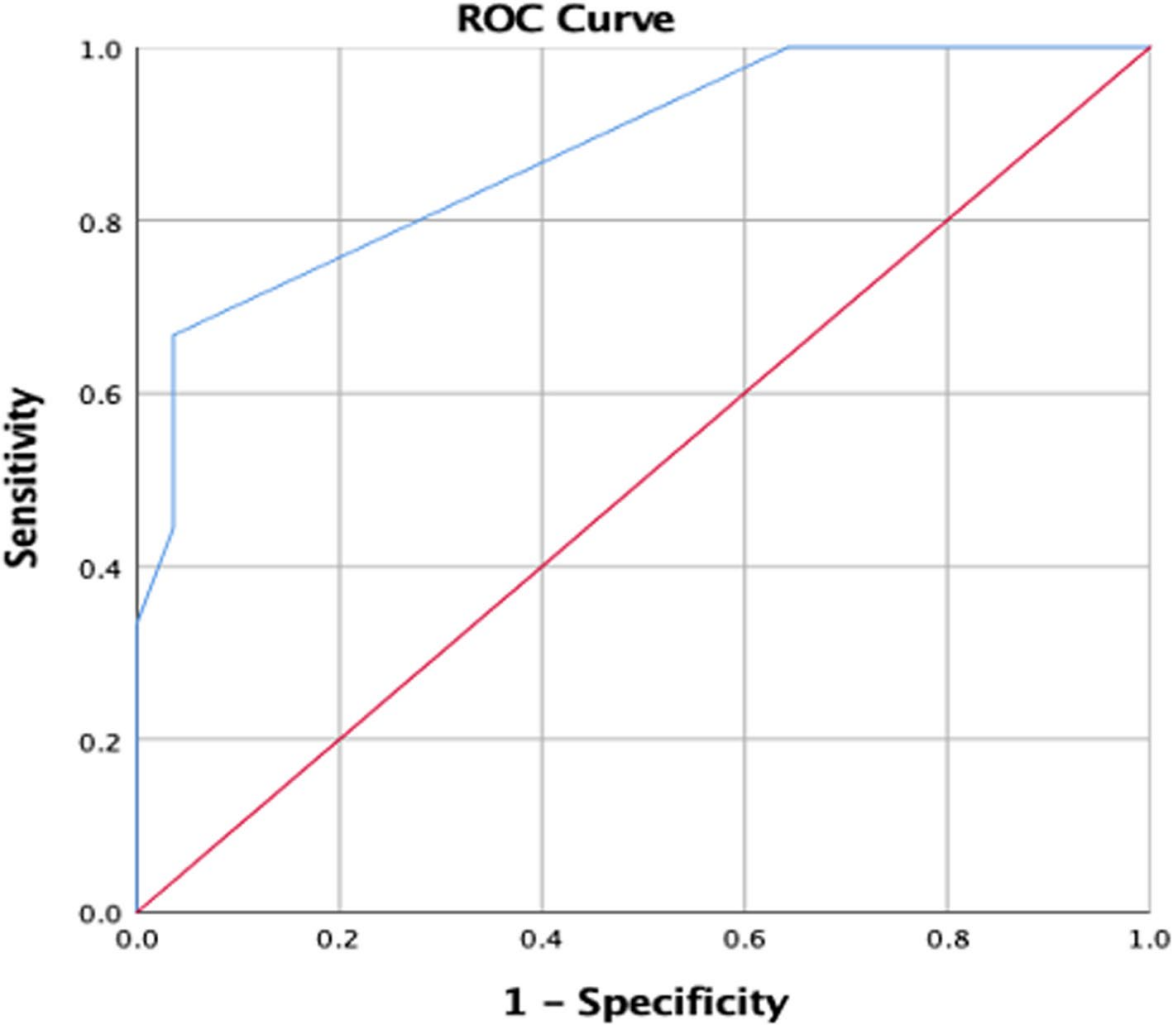
The area under the curve (AUC) was 0.877. When the vertebral body position was at 4.25, it had its highest predictive value, with a sensitivity of 66.7% and specificity of 96%

Figure 1 showed that the ROC curve had an area under the curve (AUC) of 0.877. When the vertebral body position was at 4.25, it had its highest predictive value, with a sensitivity of 66.7% and specificity of 96% (Fig. 2).

### Postnatal outcome after confirmed tethered spinal cord syndrome

In our study, six foetuses with prenatally diagnosed closed spinal dysraphism were live births. Among five infants who underwent surgical release, one had a dermal sinus, one had a lipoma, and one still required intermittent catheterisation postoperatively (Table 4).

**Table 4:** Summary of six infants prenatally diagnosed with closed spinal dysraphism/tethered cord syndrome

Cases	Postnatal imaging	Postnatal management	Postnatal development
Case1	Postal MRI detected CM at L4 and dermal cyst One month after birth	Surgical release + excision of the dermal cyst sinus two months after birth	Seven months old, with normal development
Case2	Postnatal MRI detected CM at the L3-L4 level Unknown MRI examination date	Surgical release at 11 months after birth	16 months old, with slightly delayed gross motor function and paediatric recommendation for observation
Case3	Postnatal MRI detected CM at L4 One month after birth	Surgical release one month after birth	Ten months old, with normal development
Case4	Postnatal MRI suggested a borderline low CM unknown details	Expectant no surgery	20 months old with normal development
Case5	Postnatal MRI detected lipoma and unknown CM position, Two months after birth, suggested tethered spinal cord, location unknown	Surgical release + lipoma removal at two months after birth	18 months old, requires intermittent catheterisation
Case6	Postnatal MRI detected low CM, unknown details	Surgical release five months after birth	28 months old, with normal development

## Discussion

In our study, 24.3% (9/37) of low-lying CM were complicated with spine abnormality (3 cases of open spina bifida and 6 cases of tethered cord syndrome). When the CM was between L4 and L5, its AUS to predict spinal dysraphism was 0.877, with a sensitivity of 66.7% and a specificity of 96%.

Between the 3rd and 4th week of embryogenesis, the CNS develops from the neural plate, followed by progressive folding, closure of the neural tube and separation from the ectoderm. On the 27^th^ day after fertilisation, the caudal end of the neural tube closes. By day 38, the caudal segment eventually becomes the end of the conus medullaris, the terminal filament, and the terminal ventricle (a local expansion of the central canal of the conus medullaris) [7]. The CM refers to a structure at the lower end of the spinal cord, which tapers to a cone-like shape. During the first three months of embryonic development, the spine is almost equal in length to the spinal cord. Then the spine extends caudally and grows faster than the spinal cord. Since the upper end of the spinal cord is attached to the medulla oblongata, it visually seems that the spinal cord is moving upward compared to the spine [8]. Consequently, the positions of the conus medullaris and its corresponding vertebral bodies change throughout pregnancy.

Lu et al. found that the foetal CM rose rapidly to the L3 level by 21 weeks and then rose slowly to L2-L3 by 28 weeks, according to data from 828 normal foetuses at 17–39 weeks gestation. Despite the large sample, this study may not reflect the accurate level of the foetal spinal conus due to inaccurate findings from prenatal two-dimensional (2D) ultrasound [9]. Arthurs et al. performed post-mortem MRI on 84 foetuses that died between 14 and 41 weeks of gestation. In their study, 84.2% of foetal CM was located at L4/5 or above, 22.8% were at or above the L3 before 20 weeks gestation, 50.7% were at L3 by 26 weeks, and 94.8% were at L3 by the time of birth [10].

Since The normal range of CM position has not reached a consensus, we defined a low-lying CM as being below L3, according to the ISUOG guidelines, which do not consider the upward shift of the CM with gestational age [1]. Zalel et al. conducted a prospective study that recorded the CM position in 110 normal foetuses throughout pregnancy (13–40 weeks). The team found that all foetuses' CM was at L3-L4 between 13 and 18 weeks gestation. For example, 97% were at L2-L3 between 19 and 24 weeks, and 100% were above L3 between 25 and 42 weeks [11]. In China, Li et al. also analysed 282 normal foetuses from 20 to 26 weeks of gestation and concluded that 95.5% of foetuses' CM were at L3 and above [12]. Additionally, Perlitz et al. summarised the location of the CM in 110 normal foetuses from 20 to 24 weeks of gestation. He found that 93% of foetuses' CM was above L3 and that the location of the CM did not differ with gestational age, maternal age, or foetal sex [13]. The above conclusions proved that our study's definition of low-lying conus medullaris as below L3 (excluding L3) was evidence-based.

Perlitz et al. mentioned the need for vigilance when the conus medullaris was below L3 [13]. In Li et al.'s study, the CM position of ten foetuses with spinal dysraphism was below the L3 level [12]. Our study collected data from 37 foetuses with conus medullaris below L3. Among them, 10 (27.0%) foetuses' CM was located between L3 and L4, and they had no spinal anomalies eventually. Of the 27 foetuses with CM at L4 and below, three had open spinal dysraphism, and six had closed spinal dysraphism. According to the ROC analysis, if the position of conus medullaris was at L3.75, the sensitivity and specificity to predict spinal defect were 100% and 63%, respectively. Likewise, if the position of conus medullaris was at L4.25, the sensitivity and specificity to predict spinal defect were 66.7% and 96.4%. Hence, we assume that conus medullaris at L4 and below is more suggestive of a spine defect. Nevertheless, more data should be collected to reach a more decisive conclusion.

Tethered cord syndrome is a neurological injury caused by fixation of the caudal end of the spinal cord by a hypertonic fatty infiltrated terminal filament, which in turn triggers neurological symptoms corresponding to the lower segment of the spinal cord [14]. Tethered spinal cord syndrome may occur as an isolated lesion or be associated with other lesions such as lipomas, dermal sinuses, fatty infiltration of the terminal filaments, and caudal degeneration. This study confirmed six infants with tethered cord syndrome postnatally (four isolated and two with dermal sinus and lipoma, respectively). Postnatal neurosurgical consultation and early neonatal surgery will reduce the risk of neurological injury [15].

We found no evidence of a clear correlation between an isolated foetal low-lying conus medullaris and chromosomal anomalies. Several genetic syndromes involve low-lying conus medullaris, such as Pallister–Killian syndrome [16] and Russell–Silver syndrome [17]. However, these are sporadic case reports, and the genetic syndromes always involve multiple structural anomalies. Our study identified only one case of gene abnormality according to the result of an amniocentesis performed for advanced maternal age. The clinical significance of the single-copy-number variants was not clear. The foetal conus medullaris position rose to the L3 level during this woman's pregnancy follow-up, but the patient and family chose to terminate the pregnancy because of the genetic abnormality.

However, there are a few limitations to our study. Firstly, this was a retrospective analysis with a relatively small sample. Secondly, we focused on the short-term outcomes of the infants, thereby needing further follow-up for long-term neurological development and function.

## Conclusion

The second trimester finding of low CM is associated with spinal defects, mainly open spinal dysraphism and closed spinal dysraphism/tethered cord syndrome. Careful assessment of the fetal spine should be considered especially when the location of CM is lower than L4.

## Data Availability

The datasets obtained and analysed during the current study are available from the corresponding author upon reasonable request.
